# Novel positive allosteric modulators of A_2B_ adenosine receptor acting as bone mineralisation promoters

**DOI:** 10.1080/14756366.2020.1862103

**Published:** 2020-12-17

**Authors:** Elisabetta Barresi, Chiara Giacomelli, Laura Marchetti, Emma Baglini, Silvia Salerno, Giovanni Greco, Federico Da Settimo, Claudia Martini, Maria Letizia Trincavelli, Sabrina Taliani

**Affiliations:** a Department of Pharmacy, University of Pisa, Pisa, Italy; b Department of Pharmacy, University of Naples “Federico II”, Naples, Italy

**Keywords:** A_2B_ adenosine receptor, allosteric modulators, mesenchymal stem cell, bone formation, BAY60-6583

## Abstract

Small-molecules acting as positive allosteric modulators (PAMs) of the A_2B_ adenosine receptor (A_2B_ AR) could potentially represent a novel therapeutic strategy for pathological conditions characterised by altered bone homeostasis, including osteoporosis. We investigated a library of compounds (**4**-**13**) exhibiting different degrees of chemical similarity with three indole derivatives (**1**-**3**), which have been recently identified by us as PAMs of the A_2B_ AR able to promote mesenchymal stem cell differentiation and bone formation. Evaluation of mineralisation activity of **4**-**13** in the presence and in the absence of the agonist BAY60-6583 allowed the identification of lead compounds with therapeutic potential as anti-osteoporosis agents. Further biological characterisation of one of the most performing compounds, the benzofurane derivative **9**, confirmed that such a molecule behaves as PAM of the A_2B_ AR.

## Introduction

Osteoporosis is a widespread systemic and chronic disorder characterised by decreased bone mass and micro-architectural deterioration, which predisposes to low-energy fractures. It affects the health of many adults, with an incidence that increases with age and varies among genders, being the postmenopausal women at particularly high risk^1^^,^^2^.

Several approaches have been proposed for the management of osteoporosis, in terms of prevention and/or treatment, all finalised to the achievement of normal peak bone mass, including an adequate dietary regimen, calcium and vitamin D supplementation. Among the pharmacological agents, bisphosphonates, calcitonin, oestrogens, inhibitors of receptor activator of nuclear factor κB ligand, parathyroid hormone and parathyroid hormone–related peptide should be mentioned, all possessing one or more drawbacks in terms of efficacy, safety, bioavailability, drug–drug interactions and costs^3–5^.

The increasing knowledge about the numerous signalling pathways involved in bone and osteocytes, as well as osteoblasts and osteoclasts turnover, has encouraged research projects aimed to investigate innovative therapeutic options for the treatment of osteoporosis.

In this line, emerging evidences support the role of extracellular adenosine in bone homeostasis^6–8^. Adenosine is ubiquitous and regulates several physiological and pathological conditions through the activation of specific G-protein coupled receptors, namely the A_1_, A_2A_, A_2B_ and A_3_ adenosine receptors (ARs)^9^. The binding of adenosine to its receptors causes the activation of different intracellular signalling pathways such as those involving cAMP, calcium or potassium channels, phospholipase C, phospholipase D and the mitogen-activated protein kinases^9–12^. The modulation of these pathways significantly affects the mesenchymal stem/stromal cell (MSC) biology by controlling their fates^13^.

MSCs are multipotent cells able to differentiate into osteoblasts or adipocytes. MSCs express various AR subtypes based on the differentiation lineage and stages^13^^,^^14^. Human bone marrow-derived MSCs (BM-MSCs) are the main sources for newly osteoblasts in bone. The extracellular adenosine acts as an autocrine/paracrine hormone by promoting the differentiation of MSCs to mature osteoblasts which results from activation of the A_2B_ AR^15–17^. The role of A_2B_ AR in osteoblast differentiation has been confirmed by experiments demonstrating that the deletion of this receptor in a mouse model produces a defective bone formation^18^. In addition, the A_2B_ AR negatively controls the MSCs differentiation to adipocyte^19^.

In 2013 and 2014, we disclosed three indole derivatives (**1**, **2** and **3**, Table 1) acting as positive allosteric modulators (PAMs) of the A_2B_ AR^20–22^. None of these compounds bind with appreciable affinity to the orthosteric site of each of the ARs. Moreover, they increase efficacy of adenosine or synthetic agonists without affecting their potency. To our knowledge, compounds **1**, **2** and **3** are still the only PAMs of A_2B_ AR reported in literature^20^^,^^21^.

**Table 1.** Structures of the reference indoles **1**-**3** ^20^^,^^21^ and novel compounds **4–13**.

Subsequent studies on **2**, chosen as the most promising of the three, according to *in vitro* experiments^20^^,^^21^, revealed that this compound promotes MSC differentiation and bone formation by inducing the differentiation of BM-MSCs to osteoblasts^16^.

These data suggest that the use of small molecules able to produce a positive A_2B_ AR allosteric modulation could potentially represent a novel therapeutic strategy not only for osteoporosis but also for all the pathological conditions characterised by an excessive bone destruction and/or a decrease in bone formation (e.g. fracture mal-union, osteogenesis imperfecta, rheumatoid arthritis and multiple myeloma), as well as in pathology characterised by a high bone fragility (e.g. congenital insensitivity to pain, CIP)^23^.

These results prompted a deep investigation to identify new lead compounds, structurally related to **1**, **2** and **3**, endowed with the ability to allosterically enhance A_2B_ AR agonists and to stimulate matrix mineralisation when used alone or in the presence of the orthosteric agonist BAY60-6583. In this respect, the present study was aimed at gaining information about the pharmacophoric requirements of this novel class of mineralisation agents and hopefully identifying new compounds with improved activity as compared to **2**. Thus, we selected ten compounds, whose structures are reported in Table 1, exhibiting different degrees of chemical similarity with the reference indole derivatives **1**, **2** and **3**.

The library includes novel indole-based derivatives featuring modified substitution patterns at 1- and/or 3-positions, as well as compounds in which the indole scaffold was replaced by different aromatic heterocycles. Specifically, compounds **4**-**7** feature side chains at 3-position of the indole nucleus corresponding to those of **2** (*N*-phenylglyoxylylamide) or **1** (*N*-benzylglyoxylylamide), whereas compounds **12** and **13** feature the phenylglyoxylyl side chain present in **3**. In compounds **4**-**7** and **12**-**13** the indole nucleus was maintained and was decorated with a phenylethyl (**4**, **5** and **12**) or a methyl (**6**, **7** and **13**) moiety at 1-position in place of the benzyl group of **1**-**3**; instead, in derivatives **8**-**11** the indole was replaced by different heterocycles such as benzothiophene (**8**), benzofurane (**9**), thiophene (**10**) and *N*-methylpyrrole (**11**). With the exception of the commercially available **11**, the above compounds were easily prepared by means of experimental protocols modified according to reported procedures or set up ex novo in order to obtain the desired products with high yield and purity degree.

The present paper describes the evaluation of compounds **4**–**13** as novel bone mineralisation agents and the resulting structure-activity relationships.

## Materials and methods

### Chemistry

Uncorrected melting points were determined using a Reichert Kofler hot-stage apparatus. NMR spectra were obtained on a Bruker AVANCE 400. The coupling constants (J) are given in Hertz. Magnesium sulphate was used as the drying agent. Evaporations were made *in vacuo* (rotating evaporator). Analytical TLC have been carried out on Merck 0.2 mm precoated silica gel aluminium sheets (60 F-254). Silica gel 60 (230–400 mesh) was used for column chromatography. Purity of the target inhibitors **4**–**13** was determined, using a Shimadzu LC-20AD SP liquid chromatograph equipped with a DDA Detector (λ = 254 nm) using a column C18 (250 mm × 4.6 mm, 5 µm, Shim-pack); the mobile phase, delivered at isocratic flow, consisted of acetonitrile (60-70%) and water (40-30%); flow rate 1 ml/min. All the compounds showed percent purity ≥ 95%.

Reagents, starting materials and solvents were purchased from commercial suppliers and used as received. The *N*-phenyl-indol-3-ylglyoxylamide **16**, *N*-benzyl-indol-3-ylglyoxylamide **17** were prepared according to a reported procedure^24^. Compound **11** is commercially available (Ambinter); its structure was confirmed by ^1^H-NMR and ^13 ^C-NMR.

#### N-Benzyl-2-(1-methyl-1H-pyrrol-2-yl)-glyoxylamide (11)

^1^H-NMR (DMSO-*d_6_*, ppm): 3.92 (s, 3H); 4.39 (s, 2H); 6.19-6.21 (*m*, 1H); 7.26–7.36 (*m*, 7H); 9.22 (bs, 1H). ^13 ^C-NMR (DMSO-d_6_, ppm): 37.60; 42.41; 109.54; 124.75; 127.39; 127.68; 127.76; 128.80; 134.77; 139.34; 164.74; 178.74.

#### General procedure for the synthesis of N-(indol-3-yl)glyoxylamides 4-7 and 3-(phenylglyoxylyl)indoles 12-13

Sodium hydride (60% dispersion in mineral oil, 44 mg, 1.10 mmol) was portionwise added to an ice-cooled solution of **16**,**17**^24^ or **26** (1.10 mmol) in 5 ml of anhydrous DMF. The resulting mixture was stirred at room temperature for 1 h. Then, the appropriate halide ((2-bromoethyl)benzene for **4**, **5** and **12** or methyl iodide for **6**, **7** and **13**, 1.10 mmol) was added and stirring was continued overnight. The reaction mixture was diluted with water and ice and the formed precipitate was collected by filtration and purified by recrystallization from ethanol (for **4**-**7**) or by flash chromatography (for **12**-**13**, petroleum ether 60–80 °C/ethyl acetate in ratio 8:2 as eluting system).

#### N-Phenyl-2-(1-phenylethyl-1H-indol-3-yl)glyoxylamide (4)

Yield: 61%; mp 139–140 °C; ^1^H-NMR (DMSO-*d*_6_, ppm): 3.14 (*t*, 2H, *J* = 7.4 *Hz*); 4.59 (*t*, 2H, *J* = 7.4 *Hz*); 7.19-7.21 (*m*, 2H); 7.26-7.32 (*m*, 4H); 7.34-7.41 (*m*, 4H); 7.71-7.74 (*m*, 1H); 7.87 (d, 2H, *J* = 8.0 *Hz*); 8.31-8.33 (*m*, 1H); 8.75 (*s*, 1H); 10.67 (*s*, 1H). ^13 ^C-NMR (DMSO-*d*_6_, ppm): 36.04; 48.28; 111.43; 111.84; 120.69; 121.95; 123.53; 124.11; 124.71; 127.00; 127.20; 128.84; 129.20; 129.33; 136.74; 138.40; 138.53; 141.59; 162.74; 182.05.

#### N-Benzyl-2-(1-phenylethyl-1H-indol-3-yl)glyoxylamide (5)

Yield: 67%; mp 83–85 °C; ^1^H-NMR (DMSO-*d*_6_, ppm): 3.12 (t, 2H, *J* = 7.4 *Hz*); 4.42 (d, 2H, *J* = 6.4 *Hz*); 4.56 (*t*, 2H, *J* = 7.4 *Hz*); 7.18–7.37 (*m*, 12H); 7.69–7.71 (*m*, 1H); 8.26–8.28 (*m*, 1H); 8.75 (*s*, 1H); 9.29 (*t*, 1H, *J* = 6.4 *Hz*). ^13 ^C-NMR (DMSO-*d*_6_, ppm): 36.01; 42.53; 48.23; 111.64; 111.72; 122.01; 123.37; 123.97; 126.99; 127.26; 127.35; 127.82; 128.78; 128.83; 129.31; 136.60; 138.41; 139.48; 141.54; 163.92; 182.06.

#### N-Phenyl-2-(1-methyl-1H-indol-3-yl)glyoxylamide (6)

Yield: 82%; mp 167–169 °C, lit ref^25^ mp 175–177 °C.

#### N-Benzyl-2-(1-methyl-1H-indol-3-yl)glyoxylamide (7)

Yield: 71%; mp 147–149 °C, lit ref^25^ mp 147–148 °C.

#### 1-(1-Phenylethyl-1H-indol-3-yl)-2-phenyl-1,2-ethanedione (12)

Yield: 71%; mp 101–103 °C; ^1^H-NMR (DMSO-*d*_6_, ppm): 3.08 (*t*, 2H, *J* = 7.2 *Hz*); 4.52 (*t*, 2H, *J* = 7.2 *Hz*); 7.10-7.22 (*m*, 5H); 7.34–7.40 (*m*, 2H); 7.60–7.64 (*m*, 2H); 7.74–7.88 (*m*, 2H); 7.93 (d, 2H, *J* = 7.2 *Hz*); 8.07 (*s*, 1H); 8.24 (d, 1H, *J* = 6.8 *Hz*). ^13 ^C-NMR (DMSO-*d*_6_, ppm): 35.82; 48.27; 111.94; 111.99; 121.88; 123.65; 124.38; 125.94; 126.94; 128.75; 129.25; 129.63; 130.15; 133.31; 135.19; 137.26; 138.30; 140.85; 188.58; 194.36.

#### 1-(1-Methyl-1H-indol-3-yl)-2-phenyl-1,2-ethanedione (13)

Yield: 72%; mp 88–90 °C; lit ref^26^ mp 91–92 °C.

#### General procedure for the synthesis of 2-(benzothien-3-yl)glyoxylamide 8 and 2-(aryl-2-yl)glyoxylamides 9-10

An excess of thionyl chloride (0.28 ml, 3.86 mmol) was added at 0 °C to a suspension of the acids **18**-**20** (1.93 mmol) in dry toluene (2 ml). The mixture was refluxed for 3 h and then the excess of thionyl chloride was distilled off under reduced pressure and the residue was washed three times with dry toluene. The oily residue obtained was dissolved in 10 ml of anhydrous THF, cooled at 0 °C and added with triethylamine (0.29 ml, 2.12 mmol). Then, a solution of the appropriate amine (benzylamine for **8** and **10**, 4-chlorobenzylamine for **9**, 1.93 mmol) in 2 ml of dry THF was added. The reaction mixture was left under stirring at room temperature for 24–48 h (TLC analysis; petroleum ether 60–80 °C/ethyl acetate in ratio 7:3 as eluting system). After filtering off the triethylamine hydrochloride, the solution was concentrated to dryness. The residue was triturated with diluted hydrochloric acid and then with saturated sodium hydrogen carbonate aqueous solution, washed with water and collected to give a crude product, which was purified by recrystallization from the appropriate solvent.

#### N-Benzyl-2-(benzothien-3-yl)glyoxylamide (8)

Yield: 75%; mp 87–89 °C (petroleum ether 60–80 °C); ^1^H-NMR (DMSO-*d*_6_, ppm): 4.49 (d, 2H, *J* = 6.0 *Hz*); 7.27–7.30 (*m*, 1H); 7.33-7.37 (*m*, 4H); 7.51 (*t*, 1H, *J* = 7.2 *Hz*); 7.57 (*t*, 1H, *J* = 7.2 *Hz*); 8.15 (d, 1H, *J* = 8.0 *Hz*); 8.63 (d, 1H, *J* = 8.0 *Hz*); 9.34 (*s*, 1H); 9.52 (bs, 1H). ^13 ^C-NMR (DMSO-*d*_6_, ppm): 42.65; 123.59; 124.76; 126.19; 126.63; 127.49; 127.89; 128.86; 130.21; 136.93; 139.06; 139.63; 146.24; 164.14; 183.92.

#### N-(4-Chlorobenzyl)-2-(benzofuran-2-yl)glyoxylamide (9)

Yield: 64%; mp 137–139 °C (ethyl acetate); ^1^H-NMR (DMSO-*d*_6_, ppm): 4.45 (d, 2H, *J* = 6.4 *Hz*); 7.36-7.42 (*m*, 5H); 7.59-7.63 (*m*, 1H); 7.76 (d, 1H, *J* = 8.4 *Hz*); 7.93 (d, 1H, *J* = 7.6 *Hz*); 8.35 (*s*, 1H); 9.61 (*t*, 1H, *J* = 6.4 *Hz*). ^13 ^C-NMR (DMSO-*d*_6_, ppm): 42.05; 112.75; 121.32; 124.81; 125.07; 127.25; 128.78; 129.79; 130.21; 132.09; 137.96; 149.64; 155.98; 161.83; 177.48.

#### N-Benzyl-2-(thien-2-yl)glyoxylamide (10)

Yield: 65%; mp 95.6–95.8 °C, lit. ref^27^ mp 93–94 °C (petroleum ether 60–80 °C).

#### 1-(1H-Indol-3-yl)-2-phenyl-1,2-ethanedione (26)

Thionyl chloride (0.95 ml, 13.1 mmol) and phenylglyoxylic acid **24** (2.00 g, 13.1 mmol) in dry CH_2_Cl_2_ (15 ml) were added dropwise to a cooled solution of DMAP (1.628 g, 13.1 mmol) in the same solvent (15 ml). The resulting mixture was allowed to warm at room temperature and stirred for 3 h. DMAP (1.628 g, 13.1 mmol) and indole (**14**) (1.523 g, 13.1 mmol) in anhydrous CH_2_Cl_2_ (5 ml) were then added to the cooled mixture. The reaction mixture was kept refluxing for 2 h, added with water (20 ml) and extracted with CH_2_Cl_2_ (3 × 30 ml). The combined organic phases were dried (Na_2_SO_4_), filtered and evaporated to dryness. The crude product was finally purified by flash chromatography (petroleum ether 60-80 °C/ethyl acetate in ratio 8:2 as eluting system). Yield: 65%; mp 191–192 °C, lit ref^28^ mp 194–195 °C.

### Adenosine receptor binding assay

[^3^H]DPCPX and [^3^H]NECA were obtained from DuPont-NEN (Boston, MA). ADA (Cat. N. 10102105001) was from Sigma Aldrich (Italy). All other reagents were from commercial sources and of the highest commercially available grade. CHO cells stably expressing human A_1_, A_2A_, A_3_ and A_2B_ ARs were kindly supplied by Prof. K. N. Klotz, Wurzburg University, Germany. MSCs (Cat. N. SCC034) were purchased by Sigma Aldrich (Milan, Italy).

### Human A_1_ adenosine receptors

Competition binding experiments were performed as previously reported^29^. Briefly, membranes of A_1_ CHO cells were incubated at 25 °C for 180 min in 500 µL of buffer containing [^3^H]DPCPX (3 nM) and different concentrations of the compounds. R-PIA (50 µM) was used for the determination of non-specific binding. The dissociation constant (K_d_) of [^3^H]DPCPX in A_1_ CHO cell membranes was 3 nM.

### Human A_2A_ adenosine receptors

Competition binding experiments were performed as previously reported^29^. Briefly, membranes of A_2A_ CHO cells were incubated at 25 °C for 90 min in 500 µL of buffer in the presence of [^3^H]NECA (30 nM) and six different concentrations of the newly synthesised compounds. NECA (100 µM) was used for the determination of non-specific binding. The dissociation constant (K_d_) of [^3^H]NECA in A_2A_ CHO cell membranes was 30 nM.

### Human A_3_ adenosine receptors

Competition binding experiments were performed as previously reported^30^. Briefly, cell membranes of A_3_ CHO cells were incubated at 25 °C for 180 min in 500 µL of buffer in the presence of [^3^H]NECA (30 nM) and six different concentrations of the newly synthesised compounds. NECA (100 µM) was used for the determination of non-specific binding.

### Measurement of cyclic AMP levels in A_2B_ AR CHO

Intracellular cyclic AMP (cAMP) levels were measured using a competitive protein binding method, as previously reported^31^ A_2B_ AR CHO cells were seed at a density of 24 000 cell/cm^2^ in a 24 multiwell plate. After 48 h, the medium was removed and the cells were incubated at 37 °C for 15 min with 500 µL of Dulbecco’s Modified Eagle Medium (DMEM) in the presence of ADA (1 U/mL) and the phosphodiesterase inhibitor Ro20-1724 (20 µM).

The agonist efficacy profile of the compounds at ARs was evaluated by assessing their ability to modulate intracellular cAMP levels in the absence of standard agonists. The antagonism profile of the new compounds was evaluated by assessing their ability to inhibit 100 nM NECA-mediated accumulation of cAMP. The new compounds were incubated 10 min before the addiction of the agonists. Then, cells were incubated in the reaction medium for 15 min at 37 °C. The reaction was stopped by the removal of the medium and the addition of 0.4 N HCl. After 30 min, lysates were neutralised with 4 N KOH and the suspension was centrifuged at 800 *g* for 5 min. For the determination of cAMP production, bovine adrenal cAMP-binding protein was incubated with [^3^H]cAMP (2 nM) at 0 °C for 150 min in a total volume of 300 µL. Bound radioactivity was separated by rapid filtration. The radioactivity was measured by liquid scintillation spectrometry.

### Cell cultures

Human bone marrow derived MSCs were cultured in normal growth medium (MSCGM, Sigma Aldrich), plated (5.000 cells/cm^2^) in 75-cm^2^ flasks and incubated at 37 °C in 5% CO_2_ and 95% air. The medium was changed to remove non adherent cells every 3 to 4 days and the cells were used at passages 2 to 6.

### Quantification of mineralisation

MSCs were seeded at a density of 3300 cells/cm^2^ and maintained in growth medium for 2 days. Then, medium was replaced with Mesenchymal Stem Cell Osteogenic Differentiation Medium (Cat. N. C-28013, Sigma Aldrich) and treated in the absence (control) or in the presence of BAY60-6583 (5 nM) alone or in combination with the new compounds. Treatments were repeated every 3 days and the mineralisation was quantified after 15 days of treatment. The rate of mineralisation was quantified using alizarin red staining as previously reported^32^. Briefly, cells were washed with PBS, fixed (4% formaldehyde in PBS) for 15 min and washed three times with PBS. Then, Alizarin Red S (1:100 in distilled water, adjusted to pH 4.2 and filtered) was added and incubated for 20 min, at the end cells were washed (five times) in 50% ethanol and air dried. For quantification, cells were destained overnight in 10% (w/v) cetylpyridinium chloride at room temperature and the absorbance was read using a spectrophotometer (Victor Wallac 2, Perkin Elmer) at 562 nm.

### Statistical analysis

GraphPad Prism (version 6.00) was used for data analysis and graphic presentation. Data are reported as the means ± standard errors of the means (SEM). Differences were considered statistically significant for *p* values of <0.05.

## Results and discussion

### Chemistry

**Scheme 1.**
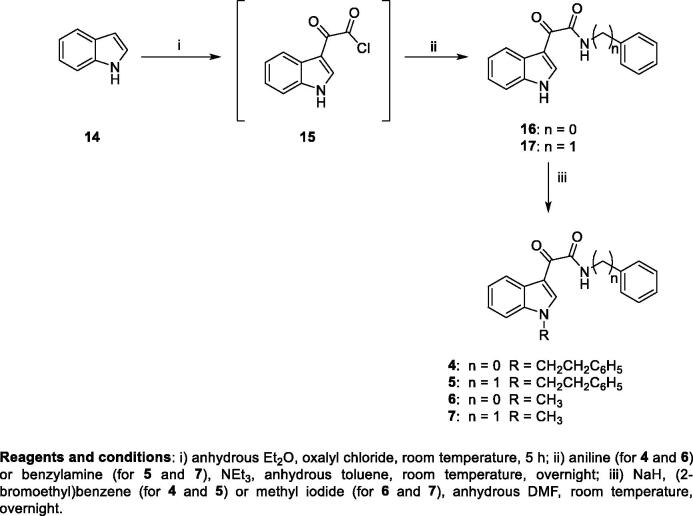
Synthesis of target compounds **4–7**. Scheme 2. Synthesis of target compounds **8–10**. Scheme 3. Synthesis of target compounds **12–13**.

The synthesis of compounds **4**-**7** was carried out following our previously procedure reported for **6** and **7**, with slight modifications^25^ (Scheme 1). Acylation of indole **14** with oxalyl chloride, in anhydrous diethyl ether, at room temperature, yielded the indol-3-ylglyoxyl chloride **15**, which was directly allowed to react overnight with the appropriate amine (aniline or benzylamine), in the presence of triethylamine in dry toluene solution at room temperature, to obtain the amides **16**, **17** (Scheme 1)^24^. Treatment of **16** and **17** with sodium hydride and subsequent addition of the appropriate halide (2-bromoethyl)benzene (for **4** and **5**) or methyl iodide (for **6** and **7**) in dry DMF yielded the target derivatives **4**-**7**, finally purified by recrystallization from ethanol.

**Scheme 2.**
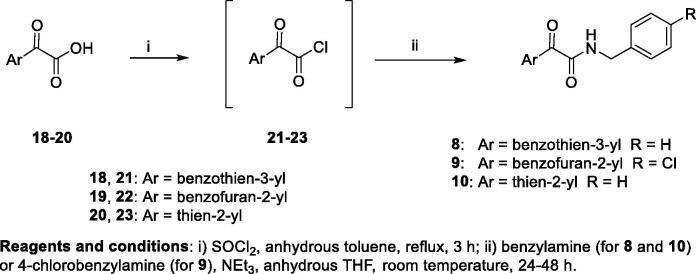
Synthesis of target compounds **4–7**. Scheme 2. Synthesis of target compounds **8–10**. Scheme 3. Synthesis of target compounds **12–13**.

Target compounds **8**-**10** were prepared according to the experimental procedure outlined in Scheme 2. The acid chlorides **21**-**23** were prepared by treating the commercially available glyoxylic acids **18**-**20** with an excess of thionyl chloride in refluxing toluene and used in the following reaction without further purification. The condensation of **21**-**23** with the appropriate amine (benzylamine for **8** and **10**, 4-chlorobenzylamine for **9**) in dry THF solution, in the presence of triethylamine, yielded the target derivatives **8**-**10**, finally purified by recrystallization from the appropriate solvent.

**Scheme 3.**
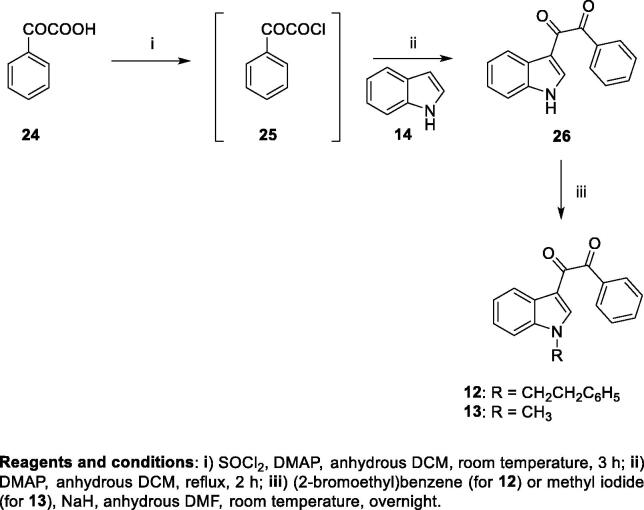
Synthesis of target compounds **4–7**. Scheme 2. Synthesis of target compounds **8–10**. Scheme 3. Synthesis of target compounds **12–13**.

Scheme 3 reports the synthesis of compounds **12** and **13**. Phenylglyoxylic acid **24** was reacted with thionyl chloride in dry dichloromethane, in the presence of DMAP, for 3 h at room temperature to provide the 2-oxo-2-phenylacetyl chloride **25** that was then treated with **14** at 50 °C for 2 h, using DMAP as a base, to yield the desired α-diketo derivative **26**. Treatment of **26** with sodium hydride and subsequent addition of the appropriate halide ((2-bromoethyl)benzene for **12** or methyl iodide for **13**) in dry DMF yielded the target derivatives **12** and **13**, finally purified by flash chromatography.

### Effects of 4-13 on MSCs differentiation to osteoblasts

**Figure 1.**
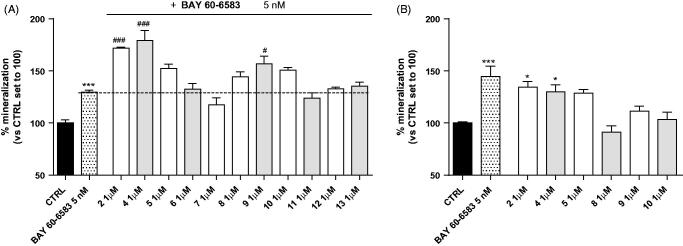
Effects of the new compounds on mineralisation mediated by MSCs-derived osteoblast. MSCs were cultured for 15 days in osteogenic medium with compounds at 1 μM concentration in the presence (A) or in the absence (B) of the A_2B_ AR agonist BAY60-6583 (5 nM). After treatments, cells were stained with Alizarin Red and mineralisation was evaluated as reported in Experimental section. Data are expressed as percentage of mineralisation versus the CTRL set to 100% and represent the mean ± SEM of at least two independent experiments. Each experiment was performed in duplicate. *p < 0.05; **p < 0.01; ***p < 0.001 vs. CTRL. #*p* < 0.05; ###*p* < 0.001 vs. BAY60-6583 or NECA.

The A_2B_ AR plays a pivotal role in MSCs differentiation to osteoblasts leading to the increase of mineralisation process. Herein, we investigated whether a series of newly synthetised compounds (**4**-**13**), potentially able to allosterically modulate the A_2B_ AR, could stimulate matrix mineralisation when used in the presence of the orthosteric agonist BAY60-6583 or alone (Figure 1).

To evaluate the mineralisation activity of compounds **4**-**13**, they were first assayed in the presence of BAY60-6583 at 5 nM concentration (Figure 1(A)). Such a concentration was selected based on the reported potency of BAY60-6583 on MSCs (EC_50_ = 6.0 nM)^16^. Under these experimental conditions it can be safely assumed that the orthosteric site is mainly occupied by BAY60-6583 rather than adenosine, owing to the much higher potency and concentration of the synthetic agonist compared with the natural transmitter present in the culture medium at physiological concentrations^33^. The mineralisation activity was expressed as percentage with respect to 100% assigned to the control (measurements in absence of BAY60-6583). The activity of the parent compound **2** in the presence of 5 nM BAY60-6583 (171.9 ± 0.8% *p* < 0.001 vs BAY60-6583) served as a benchmark. MSCs, cultured under osteogenic conditions, underwent a spontaneous, time-dependent, mineralisation^16^. Accordingly, cells treatment with BAY60-6583 induced a significant increase in the mineralisation process of MSCs (129.5 ± 1.9% *p* < 0.001 vs control; Figure 1(A)). In this preliminary test, some new derivatives potentiated BAY60-6583 activity (Figure 1(A)), thanks to their hypothesised properties of positive allosteric modulators of A_2B_ AR: namely **4** (179.2 ± 9.6%. *p* < 0.001 vs BAY60-6583), **5** (152.3 ± 4.3%), **8** (144.4 ± 4-9%), **9** (156.8 ± 7.6%, *p* < 0.05 vs BAY60-6583) and **10** (150.6 ± 2.6%). Among these, only derivatives **4** and **9** significantly ameliorated BAY60-6583 activity, while for compounds **5**, **8** and **10** the increase is not statistically significant. It is worth noting that **4** is more active than the reference compound **2**.

The activity of compound **9** suggests that the indole scaffold together with a *N*-arylalkyl substituent (benzyl or 2-phenylethyl) at 1-position are probably not key pharmacophore elements to display positive allosteric modulation of the A_2B_ AR. Of course, such a conclusion should be supported by further experiments demonstrating the precise nature of the events occurring at the molecular levels. The activity measured in the mineralisation assay reflects, in fact, not only pharmacodynamic factors but also pharmacokinetic phenomena, such as stability to metabolism, which cannot be taken into account separately.

The compounds able to modify the mineralisation induced by BAY60-6583 in the above described assay (**4**, **5**, **8**, **9** and **10**) were subsequently evaluated for their mineralisation activity in absence of BAY60-6583 in order to assess their therapeutic potential (Figure 1(B)). It has been demonstrated that the presence of endogenous adenosine in the medium affects MSCs differentiation^16^; thus, under these experimental conditions, the tested compounds alone could potentiate the adenosine activity mediated by the A_2B_ AR activation, acting as PAMs. In this assay, those compounds displaying a mineralisation activity higher than that of the control (set to 100%) were considered active. Three compounds revealed to be more active than the control, specifically **4** significantly enhanced the mineralisation with respect to the control (130.0 ± 6.8%, *p* < 0.01), while compounds **5** (128.7 ± 3.6%) and **9** (111.5 ± 4.6%) displayed a mineralisation activity higher compared with the control although not significant. These products might be considered potential anti-osteoporosis agents as they are able to increase the bone mineralisation in experimental conditions close to the physio-pathological ones. The inactivity of **8** and **10** might be ascribed to their low affinity for the allosteric site of the A_2B_ AR. However, as mentioned before, we cannot rule out that factors others than the pharmacodynamic ones can interfere with the ability of **8** and **10** to act as A_2B_ AR allosteric enhancers of adenosine.

The obtained results prompted us to focalise our attention on the most promising compounds able to enhance osteoblast differentiation. However, unlike **4** and **5** that are structurally analogues of our already reported PAMs^20^^,^^21^, **9** features a peculiar chemical structure that could make it a potential lead compound for the development of novel PAMs. In this respect, a more accurate characterisation of **9** was carried out.

When assayed in radioligand binding assays, **9** displayed no appreciable binding affinity for A_1_, A_2A_ and A_3_ ARs (K_i_ > 10 000 nM). Compound **9** was then evaluated for its ability to modulate the increase in cAMP levels, either alone or in the presence of an EC_50_ concentration of the agonist NECA in human A_2B_ AR-transfected CHO cells (Table 2). When tested alone at a 10 µM concentration, **9** did not significantly increase cAMP levels, demonstrating a lack of A_2B_ AR agonist activity. Interestingly, **9** potentiated the effects of NECA, to a higher extent with respect to the parent compound **2**, suggesting that this compound may behave as PAM of the A_2B_ AR.

**Table 2.** Effects of **2** and **9** on cAMP production in CHO cells expressing human A_2B_ AR.^a^

Cpd	% cAMP production (vs NECA 100 nM set to 100%)^b^	
	***h*A_2B_ AR**	
	Alone	+ agonist
**2**	6.2 ± 1.3	150.6 ± 15.7***
**9**	2.7 ± 1.3	184.0 ± 11.7***

^a^The effect of each compound (10 µM) on cAMP production was evaluated in CHO cells expressing human A_2B_ AR (see biological section). Each compound was tested alone or in the presence of the agonist NECA (100 nM). ^b^Data are expressed as percentage of cAMP production versus agonist maximal effect set to 100%. All data represent the mean ± SEM of at least three independent experiments. ***p < 0.001 vs agonist alone.

**Figure 2.**
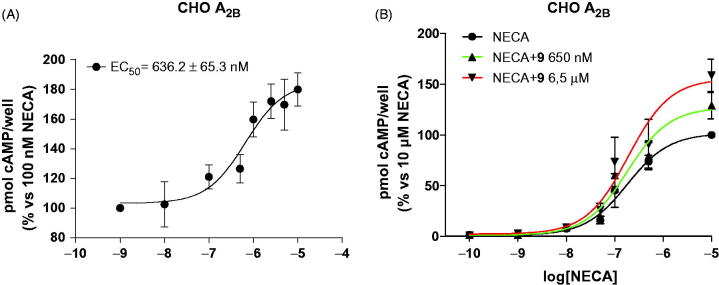
Effects of compound **9** as positive allosteric modulator of the A_2B_ AR. A) Effects of **9** on NECA-mediated cAMP accumulation in human A_2B_ AR-transfected CHO cells. CHO cells were treated with an EC_50_ NECA concentration (100 nM) in the absence or presence of different concentrations of the tested compound (1 nM–10 μM). B) Effects of **9** on agonist Emax. CHO cells were treated with different NECA concentration (1 nM–10 μM) in the absence or presence of **9** (650 nM or 6.5 μM). After 15 min incubation, the reaction was stopped and the intracellular cAMP levels were quantified. The data are expressed as the percentage of cAMP/well versus the maximal NECA effect, which was set to 100% and represent the mean ± SEM of at least three different experiments. Each experiment was performed in duplicate.

Then, the potency of **9** in modulating the activity of A_2B_ AR agonists was determined by assessing the effects of different concentrations (ranging from 1 nM to 10 µM) on cAMP accumulation induced by an EC_50_ concentration of the agonist NECA (100 nM). The resulting concentration–response curves (Figure 2(A)) indicated that **9** exhibited a positive allosteric modulation of A_2B_ AR with an EC_50_ 636.2 ± 65.3 nM. Subsequently, the effects of **9** on the potency and efficacy of NECA were evaluated by assessing agonist concentration–response curves in the absence or presence of two different concentrations of **9** (650 nM and 6.5 µM). Compound **9** significantly enhanced the efficacy of NECA in stimulating cAMP accumulation without affecting agonist potency (Figure 2(B)).

## Conclusions

A small library of molecules (**4**-**13**) exhibiting different degrees of chemical similarity with the recently identified indole-based positive allosteric modulators (PAMs) of the A_2B_ AR **1**–**3** was investigated for their potential as bone mineralisation agents. Compounds **4** and **9** turned out to stimulate matrix mineralisation in MSCs either in the presence or in the absence of the agonist BAY60-6583. The structure-activity relationships of **4**–**13** suggest that the indole scaffold and a *N*-arylalkyl substituent (benzyl or 2-phenylethyl) at 1-position are probably not key pharmacophore elements to display positive allosteric modulation of the A_2B_ AR. The biological properties of the benzofurane derivative **9**, featuring a peculiar chemical structure with respect to the reference indoles **1**–**3**, were more accurately characterised. The obtained results confirmed that such a molecule behaves as PAM of the A_2B_ AR, making it a lead structure for the development of novel compounds potentially acting as anti-osteoporosis agents.
